# The response of microbiome assembly within different niches across four stages to the cultivation of glyphosate-tolerant and conventional soybean varieties

**DOI:** 10.3389/fmicb.2024.1439735

**Published:** 2024-09-25

**Authors:** Shengqian Chao, Yu Sun, Yin Zhang, Yifan Chen, Lili Song, Peng Li, Xueming Tang, Jingang Liang, Beibei Lv

**Affiliations:** ^1^Biotechnology Research Institute, Key Laboratory of Agricultural Genetics and Breeding, Shanghai Academy of Agricultural Sciences, Shanghai, China; ^2^Key Laboratory for Safety Assessment (Environment) of Agricultural Genetically Modified Organisms, Ministry of Agriculture and Rural Affairs, Beijing, China; ^3^Shanghai Agricultural Biosafety Evaluation and Testing Professional Technical Service Platform, Shanghai, China; ^4^School of Agriculture Biology, Shanghai Jiao Tong University, Shanghai, China; ^5^Development Center of Science and Technology, Ministry of Agriculture and Rural Affairs, Beijing, China; ^6^CIMMYT-China Specialty Maize Research Center, Shanghai, China

**Keywords:** developmental stage, glyphosate, microbiome, niche, soybean, transgene

## Abstract

**Introduction:**

Plants are inherently connected with the microbiome, which plays a crucial role in regulating various host plant biological processes, including immunity, nutrient acquisition, and resistance against abiotic and biotic stresses. Many factors affect the interaction between plants and microbiome.

**Methods and results:**

In this study, microbiome samples were collected from five niches (bulk soil, rhizoplane, root endosphere, phylloplane, and leaf endosphere) across four developmental stages (seedling, flowering, podding, and maturity) of various soybean varieties. Composition and structure of bacterial and fungal communities were analyzed using 16S rRNA gene and ITS (Internally Transcribed Spacer) region amplicon sequencing. It was observed that both niches and developmental stages significantly impact on the assembly and composition of soybean microbiome. However, variety, presence of a transgene, and glyphosate application had minimal effects on microbial communities. The dominant microbiome varied across the five niches, with most containing beneficial microbial communities capable of promoting plant growth or increasing disease resistance. Types and abundance of the dominant microbes affected network stability, potentially resulting in functional changes in different ecological niches.

**Conclusion:**

This study provides theoretical evidence for microbial protection of plants against diseases and demonstrates that systematic analysis of the composition and diversity of soybean microbiomes can contribute to the development of biological control technologies.

## 1 Introduction

Natural microbial communities associated with plants include microbiomes in the phyllosphere, soil, and rhizosphere. Within these niche-specific communities, multiple pathogenic bacteria coexist with beneficial microbial groups that serve the host (Cernava et al., 2019). Beneficial plant microbiomes can promote plant growth and development through various pathways, improving plant stress resistance and adaptability (Compant et al., 2024; Avis et al., 2008). In some rhizosphere microbiomes, bacteria, such as *Rhizobia*, facilitate biological nitrogen fixation to meet plant nitrogen demands (Liu et al., 2020a). Certain microbiomes can also solubilize phosphorus-containing minerals, thereby increasing phosphorus bioavailability (Granada et al., 2018). Moreover, rhizospheric and endophytic bacteria can inhibit pathogens and improve the availability of minerals with production of phytohormones. For example, *Gluconacetobacter* can synthesize IAA (indole acetic acid) and GA (gibberellic acid) to affect root development, while gluconic acid promotes P and Zn chelation (Reis and Teixeira, 2015). Strongly acidic soils are characterized by high aluminum toxicity and low phosphorus availability, which suppress legume plant growth and nodule development, and mycorrhizae enhance soybean plant growth and aluminum stress tolerance by shaping the microbiome assembly in an acidic soil (Wen et al., 2023). When plant roots were infected by fungi, *Chitinophagaceae* and *Flavobacteriaceae* were enriched in the root endosphere, which can consistently suppress fungal root disease (Carrión et al., 2019). Phyllosphere microbiomes can promote plant growth by increasing nutrient absorption, synthesizing plant hormones, and assisting plants in adapting to abiotic stress (Vorholt, 2012; Zhan et al., 2022). In asymptomatic rice phyllosphere, native *Aspergillus cvjetkovicii* produces 2(3H)-benzofuranone toantagonize *Magnaporthe oryzae*, one of the most aggressive rice pathogens (Fan et al., 2019).

Differences in microbial diversity can also affect their functions. With the increase in microbial species diversity, functional diversity has sharply increased (Ruhl et al., 2022). Compared to bulk soil, the diversity and complexity of rhizosphere microbial communities were higher, and microbial activity and residue levels were also higher. Further analysis showed that microbial activity and residue were significantly correlated with microbial composition and symbiotic network complexity (Qiu et al., 2022). From the root sheath to the bulk soil, the types and functions of microbial communities gradually decrease, and the content of genes involved in carbon cycling, sulfur cycling, and phosphorus cycling was higher than that of microbiome in the soil (Wang et al., 2022). Phyllosphere supported the enormous diversity of bacteria, yeast, and filamentous fungi. It was precisely the diversity of these fungi and bacteria that led to functional differences, such as promoting carbon and nitrogen cycling, maintaining ecosystem productivity, and tolerance to abiotic (drought and salinity) and biotic (pathogen) stress (Bashir et al., 2022). The plant microbiome has been shown to affect the adaptability and function of host plants, and leaf bacterial diversity was positively correlated with ecosystem productivity (Laforest-Lapointe et al., 2017).

Plant niche is a major factor shaping composition of plant microbiomes (Trivedi et al., 2020). For example, bacterial operational taxonomic units (OTUs) are different in rice sprouts, roots, and stems (Wang et al., 2016), and significant differences in diversity are observed among different plant niches and developmental stages (Moroenyane et al., 2021a). In tomatoes, plant development stage influences the spatially dynamic process of microbial assembly (Cordovez et al., 2021). Moreover, maize developmental stage has substantial effects on microbial diversity, composition, and interkingdom networks within plant niches compared with those in soil, with the strongest effects on the phylloplane (Xiong et al., 2021a).

The roots of different plant genotypes can also shape microbial communities (Li et al., 2022a). In maize, correlations were detected between rhizosphere microbial communities and host genotypes, with 143 OTUs significantly regulated by plant genotype (Walters et al., 2018). Transgenic plants, considered as different genotypes, also changed microbial communities compared with WT. The phosphomannose isomerase gene *manA* plays a central role in forming the structure of extracellular polysaccharides (EPSs) and subsequent biofilms and May negatively affect the microbial diversity of plant-associated microbiota because EPS is an important virulence factor in various pathogenic bacterial strains (Amos et al., 2011; Castiblanco and Sundin, 2016). Genes encoding selective markers in transgenic plants can also disrupt the balance between symbiotic and pathogenic bacteria (Pepoyan and Chikindas, 2020). However, studies also show limited effects of transgenic crops on soil microbiomes. Two-year field experiments with transgenic soybean ZD91 revealed no significant effects on rhizosphere bacterial communities; instead, plant growth stage and year had the strongest influences (Liang et al., 2018). In rice, no significant differences in soil bacterial community structure were observed between transgenic and parental varieties, although notable differences were observed compared with non-parental varieties, suggesting limited effects of planting transgenic Bt rice on soil microbiomes (Wang et al., 2019).

Glyphosate is a widely used herbicide that can stimulate plant growth at low doses (Brito et al., 2018; Velini et al., 2008). This effect on growth is contingent on the root microbiome composition, especially the presence of root growth-inhibiting strains (Ramirez-Villacis et al., 2020). Differences in taxonomic and functional microbial diversity were detected in soils planted with traditional and transgenic soybeans resistant to glyphosate for almost a decade using the same set of herbicides, but differences were minimal compared with sites (Babujia et al., 2016). For EPSPS (5-enolpyruvylshikimate-3-phosphate synthase)-transgenic maize CC-2 and the WT at earing time or flowering stage, the *α* and *β* diversity of the root-associated bacterial community were not significantly different, although significant differences in microbial abundance were observed (Wen et al., 2019).

Soybean is an important economic crop and the main source of plant protein and oil. In recent times, researchers have been increasing their efforts to design a sustainable method to improve the production of soybean (Jordaan et al., 2019). Due to the environmental threats caused by the application of synthetic fertilizers, it has become urgent to adopt biological methods and sustainable measures to increase soybean yields. The endogenous microbiome in soybeans can increase the nitrogen pool in the soil, enhance plant nutrition, and improve productivity (Dubey et al., 2021). Therefore, it is crucial to study the factors that affect the interaction between soybean and microbiome, and how to use these factors to alter the assembly of soybean microbiome.

Despite some discoveries being made by several studies concerning microbial diversity in distinct plant niches of soybean across its various developmental stages (Gdanetz et al., 2021; Moroenyane et al., 2021a,b), our understanding of the factors influencing soybean microbiomes, the effects of beneficial microbiomes, and the identification of beneficial microbiomes remains relatively limited. However, beneficial microbiomes hold potential for increasing plant protection. In this study, we investigated the microbial diversity of bulk soil, rhizoplane, root endosphere, phylloplane, and leaf endosphere of different soybeans across different growth stages, analyzed the dominant microbiome in the different niches, examined the effects of stages, transgenes, and glyphosate on microbial communities, and explored the migration and changes in microbial communities across ecological niches. Our goal was for the findings to lay a foundation for improving plant resistance and increasing plant quality.

## 2 Materials and methods

### 2.1 Experimental design and sampling

A pot experiment was performed in the greenhouse. The seeds of soybean were provided by the Institute of Crop Sciences, Chinese Academy of Sciences. Seeds of transgenic soybean Zhonghuang6106 (T), which has resistance to glyphosate, conventional parental line Zhonghuang6106 (P), and conventional variety (CK), were sown in soil collected in Baihe base of Shanghai Academy of Agricultural Sciences (31°24′N, 121°11′E), Shanghai, China. Transgenic soybean Zhonghuang6106 was also treated with water and glyphosate (1 mg/L) (TR) in the seedling (V3) stage. The experimental design was randomized block, with three replicates for each material and three seedlings per replicate. Watered about once or twice a week, depending on soil humidity. Plants of V3, flowering (R2), podding (R5), and maturity (R8) stages were sampled in the experiment, with samples collected in five niches: bulk soil (BS), rhizoplane (R), root endosphere (RE), phylloplane (L), and leaf endosphere (LE).

### 2.2 Sample collection

For root and leaf sampling, three individual soybean plants were collected from each pot. In the laboratory, each plant was separated into root and stem samples at cotyledonary nodes; leaf samples were the leaves removed from the stem samples (total of 50–100 g fresh weight for each pot) (Xiong et al., 2021b). Bulk soil sample was collected at a depth of 12 cm (topsoil) and 20 cm from the roots, about the pot edge that did not contain any roots. Roots were gently dislodged from soil. Manual shaking removed soil attached to roots. Roots were immersed in sterile PBS (phosphate-buffered saline) solution and incubated at 180 rpm for 20 min. After incubation, the roots were removed, and sterile PBS solution was added for a second 20-min incubation. Subsequently, the roots were subjected to ultrasound washing for 10 min (parameters: 160 W, 30 s/30 s). Finally, the roots were rapidly frozen with liquid nitrogen, ground into powder, and used to extract the endophytic microbiome, which was considered as root endosphere. The three washing solutions were combined and passed through a 0.2-μm filter membrane. Filter membranes were collected, considered as rhizoplane, and stored at −80°C until analyses. The sampling method of leaf endosphere and phylloplane was the same as the root endosphere and rhizoplane.

### 2.3 DNA extraction and PCR amplification

Total microbial genomic DNA was extracted from samples stored at −80°C. The bulk soil DNA was extracted from 0.4 g soil using the FastDNA® Spin Kit for Soil (MP Biomedicals, United States). For rhizoplane and phylloplane DNA extraction, the solution of washing roots and leaves was passed through 0.2-μm filter membrane; the microbiome was all collected on the filter membrane and subjected to DNA extraction using the FastDNA® Spin Kit for Soil. Endophytic DNA was extracted from the same roots and leaves used for rhizoplane and phylloplane DNA after further surface sterilization (Xiong et al., 2021b). DNA quality and concentration were determined by 1.0% agarose gel electrophoresis and a NanoDrop2000 spectrophotometer (Thermo Scientific, United States), respectively. DNA was stored at −80°C until further use. The hypervariable region V3–V4 of the bacterial 16S rRNA gene was amplified with the primer pairs 338F (5′-ACTCCTACGGGAGGCAGCAG-3′) and 806R (5′-GGACTACHVGGGTWTCTAAT-3′) (Liu et al., 2016) by a T100 Thermal Cycler PCR thermocycler (Bio-Rad, United States). For fungal libraries, the ITS1 region was amplified using the primers ITS1-F (5′-CTTGGTCATTTAGAGGAAGTAA-3′) and ITSR (5′-GCTGCGTTCTTCATCGATGC-3′) (Yang H. J. et al., 2023; Yang Q. et al., 2023). The PCR reaction mixture contained 4 μL of 5× Fast Pfu buffer, 2 μL of 2.5 mM dNTPs, 0.8 μL of each primer (5 μM), 0.4 μL of Fast Pfu polymerase, 10 ng of template DNA, and ddH_2_O to a final volume of 20 μL. PCR amplification cycling conditions were as follows: initial denaturation at 95°C for 3 min, followed by 27 cycles of denaturing at 95°C for 30 s, annealing at 55°C for 30 s, and extension at 72°C for 45 s, and single extension at 72°C for 10 min, and end at 4°C. The PCR product was extracted from 2% agarose gel and purified using a PCR Clean-Up Kit (YuHua, Shanghai, China) according to the manufacturer’s instructions. The product was quantified using Qubit 4.0 (Thermo Fisher Scientific, United States).

Purified amplicons were pooled in equimolar amounts and paired-end sequenced on an Illumina PE300/PE250 platform (Illumina, San Diego, CA, United States) according to standard protocols by Majorbio Bio-Pharm Technology Co. Ltd. (Shanghai, China). The raw sequencing reads are deposited in the NCBI Sequence Read Archive database (Accession Number: PRJNA1092852).

### 2.4 Amplicon sequence processing and analysis

After demultiplexing, the resulting sequences were quality-filtered with fastp (0.19.6) (Chen et al., 2018) and merged with FLASH (v1.2.11) (Magoč and Salzberg, 2011). Then, the high-quality sequences were denoised using the DADA2 (Callahan et al., 2016) plugin in the Qiime2 (v2020.2) (Bolyen et al., 2019) pipeline with recommended parameters to obtain single-nucleotide resolution based on error profiles within samples. DADA2-denoised sequences are usually called amplicon sequence variants (ASVs). To minimize the effects of sequencing depth on alpha and beta diversity measures, the number of sequences from each sample was limited to 20,000, which still yielded an average Good’s coverage of 97.90%. Taxonomic assignment of ASVs was performed using the Naive Bayes consensus taxonomy classifier implemented in Qiime2 and the SILVA 16S rRNA database (v138). The metagenomic function was predicted by PICRUSt2 (Phylogenetic Investigation of Communities by Reconstruction of Unobserved States) (Douglas et al., 2020) based on ASV representative sequences. PICRUSt2 is a software containing a series of tools as follows: HMMER was used to align ASV representative sequences with reference sequences; EPA-NG and Gappa were used to place ASV representative sequences into a reference tree; the castor was used to normalize the 16S gene copies; and MinPath was used to predict gene family profiles and locate them into gene pathways. The entire analysis process followed the protocols of PICRUSt2.

### 2.5 Statistical analysis

To calculate alpha diversity indices, QIIME2 software was used to set the flattening depth to 95% of the lowest sequencing depth sample sequence in all samples, and R script was used to plot the data in box plots. LMM (linear mixed model) analysis was performed by using alpha diversity index or abundance tables at various classification levels to construct a model using the lmerTest package in R, and ANOVA was performed for verification. Neutral models used the flattened ASV/OTU table to calculate Nm and Rsqr values using Hmisc and minpack. Lm packages in R, which were then visualized using the grid system in R. Beta_NTI used unpillared ASV/OTU tables and phylogenetic tree files for community construction analysis, calculated through the R package picante and iCAMP. *β* NTI (Nearest Taxon Index) and RCBray values were used to determine whether a community was dominated by deterministic or stochastic processes. The similarity among the microbial communities in different samples was determined by principal coordinate analysis (PCoA) based on Bray–Curtis dissimilarity using the vegan v2.5–3 package (Oksanen et al., 2007). Non-metric multi-dimensional scaling (NMDS) analysis was performed using the vegan package in R. A two-dimensional sorting chart was used to describe the structural distribution of community samples, where the elliptical dashed line represented a 95% confidence ellipse. Adonis and Anosim tests were conducted using QIIME2 to verify the distribution pattern presented in a graph. PERMANOVA tests used the QZA file of the distance matrix, and QIIME2 was used to call the PERMANOVA algorithm to test the significance of differences between groups (Oksanen et al., 2007). Beta dissertation used a distance matrix file, and Bray–Curtis dissimilarity was calculated using the betadisper function in the R vegan package, analyzing whether groups were significant according to ANOVA. As in a previous study (Li et al., 2022b), the core ASV was the ASV with high abundance in all samples. Co-occurrence networks were constructed to explore the internal community relations across the samples (Barberan et al., 2012). Unperforated ASV/OTU tables were used to calculate Spearman correlation coefficients in bacterial and fungal interaction networks, with screening for relations with correlations with | *r* | > 0.6 and *p* < 0.05. For the boundary network, relations were filtered for correlations with | *r* | > 0.8 and *p* < 0.01. Visualization was created using R igraph. Source Tracker 2 (https://github.com/caporaso-lab/sourcetracker2) also used the flattened ASV/OTU table to perform traceability analysis.

## 3 Results

### 3.1 Transgene had little effect on soybean microbiome

Totals of 14,348,482 bacterial 16S rRNA and 15,700,058 fungal ITS high-quality reads were obtained from 240 samples (Supplementary Table S1). After denoising, removing low-quality and chimeric sequences with DADA2, the reads were classified into 81,482 bacterial ASVs and 10,129 fungal ASVs (Supplementary Table S1).

**Figure 1:**
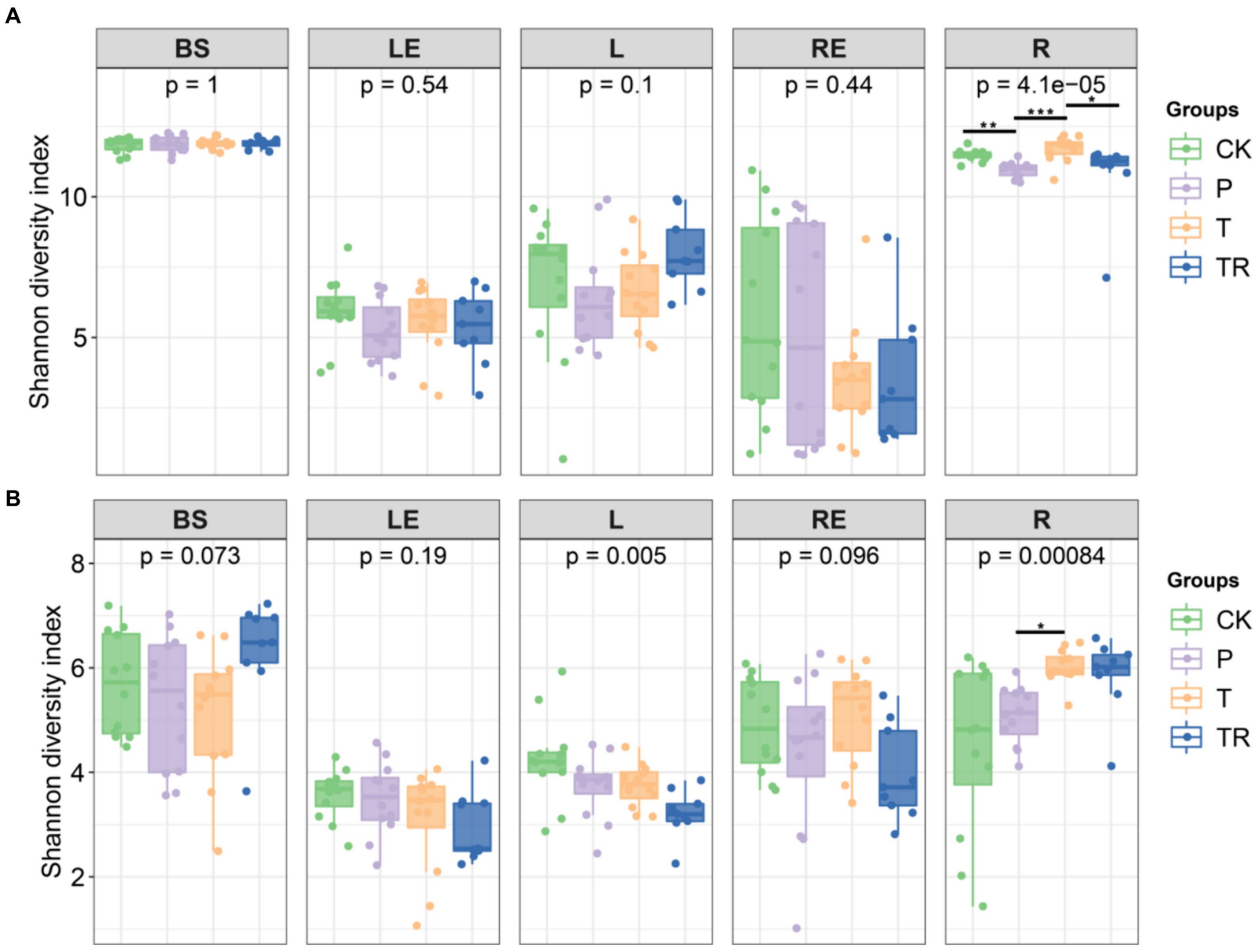
Alpha diversity of **(A)** bacterial and **(B)** fungal communities in different niches in different soybean lines (ANOVA, *n* = 3). *, **, and *** significant at 0.05, 0.01, and 0.001 probability levels, respectively. CK, conventional variety; P, conventional parental line Zhonghuang 6106; T, transgenic soybean Zhonghuang 6106; TR, transgenic soybean Zhonghuang 6106 was treated with glyphosate (1 mg/L). BS, bulk soil; L, phylloplane; LE, leaf endosphere; R, rhizoplane; RE, root endosphere.

The alpha diversity of bacterial and fungal communities in different niches was analyzed based on the Shannon diversity index and Chao1 richness. Among soybean varieties, bacterial diversity in the different niches was not significantly different, except in the rhizoplane. In rhizoplane, the Shannon diversity of bacterial communities in parental line (P) was significantly lower than conventional soybean (CK), showed variety affected the bacterial community of rhizoplane. The Shannon diversity of bacterial communities in transgenic soybean (T) was significantly higher than P, showed that the presence of a transgene also affected the bacterial community of rhizoplane. The Shannon diversity of bacterial communities in transgenic soybean which treated with glyphosate (TR) was significantly lower than T, showed that glyphosate affected the bacterial community of rhizoplane. The Shannon diversity of the bacterial communities was similar in bulk soil at different stages of soybean growth except in BS between CK and P in R8 (Supplementary Figures S1A–D). Among soybean varieties, diversity of fungal communities was only significantly affected by the transgene in the rhizoplane, with the lowest Shannon diversity in CK and the highest in T and TR (Figure 1B). Therefore, among different niches and soybean stages, the alpha diversity of bacterial and fungal communities was only significantly affected in the rhizoplane, with the highest Shannon diversity in T (Figure S1a–h).

Niche was the main factor affecting bacterial and fungal communities based on Shannon diversity index and Chao1 richness (Supplementary Table S2). Shannon diversity analysis showed stage only affected fungal community diversity, and transgene had little effect on either bacterial or fungal communities (Supplementary Table S2). Bacterial diversity was significantly different among soybean lines only in the rhizoplane (Figure 1A), with the lowest Shannon diversity in the P and the highest in T in the rhizoplane at different stages, indicating that transgene increased the diversity of the rhizoplane microbiome (Supplementary Table S1).

### 3.2 Plant niches affected the transition of bacterial and fungal communities based on neutral community model analysis

**Figure 2:**
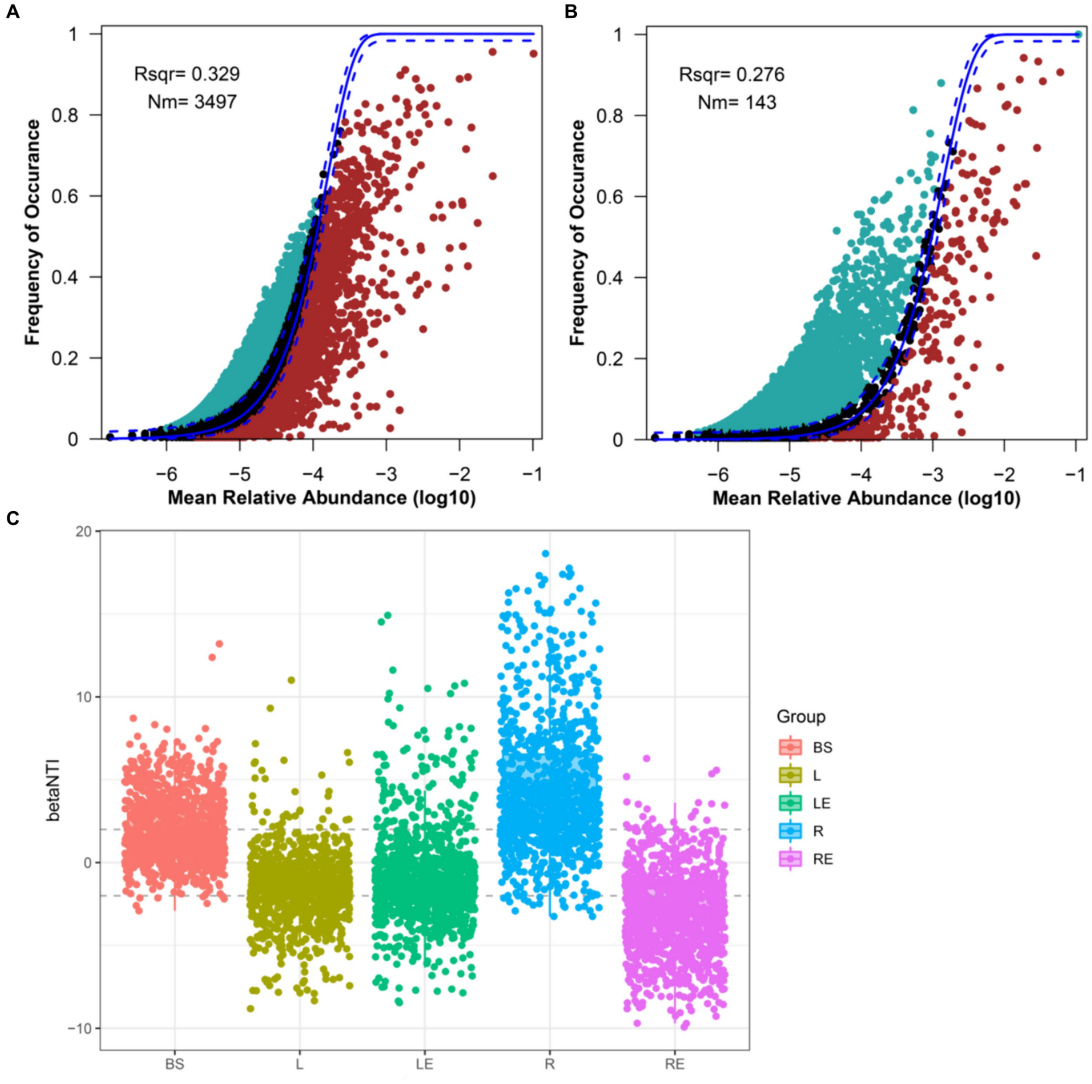
Fit of the neutral community model (NCM) of bacterial and fungal community assembly. Predicted occurrence frequencies for **(A)** bacteria and **(B)** fungi. Solid blue lines indicate the best fit to the NCM, and dashed blue lines represent 95% confidence intervals around the model prediction. ASVs that occur more or less frequently than predicted by the NCM are shown in different colors. Nm indicates the metacommunity size times immigration; Rsqr indicates the fit to NCM. **(C)** Beta_NTI evaluation of community assembly process. BS, bulk soil; L, phylloplane; LE, leaf endosphere; R, rhizoplane; RE, root endosphere.

Based on the results of alpha diversity analyses, the assembly mechanisms of bacterial and fungal communities in the five niches were analyzed using a neutral community model (NCM). The overall fit of neutral community models for bacteria and fungi was not high, but the fitting degree of the bacterial community was higher than that of the fungal community. The better fit for the bacterial community indicated that the assembly of bacterial communities, compared with that of fungi, was more affected by random processes and less affected by deterministic processes (Figures 2A,B). The degree of fit to the NCM for bacterial communities was in the order leaf endosphere < phylloplane < root endosphere < rhizoplane < bulk soil, whereas the degree of fit for fungal communities was as in the order root endosphere < phylloplane < rhizoplane < bulk soil < leaf endosphere (Supplementary Figures S2A–J). The lowest degree of fit of fungal communities was in the root endosphere, indicating that assembly of the fungal microbiome in the root endosphere was subjected to increased selection; it was speculated that it was mainly affected by the bulk soil and rhizoplane. Simultaneously, the Nm of aboveground (phylloplane and leaf endosphere) was lower than that of belowground (bulk soil, rhizoplane, and root endosphere), and changes in niche or habitat played a more critical role in the assembly of bacterial communities in soybean, while random processes dominated the assembly process of bacterial communities in the phylloplane and leaf endosphere (Figures 2A,B).

To infer the effects of different niches on the construction of community structure, beta_NTI plots were also conducted. Beta_NTI absolute values of bulk soil, phylloplane, and leaf endosphere were mainly less than 2. The results showed that community transition was mainly controlled by selective processes in bulk soil, phylloplane, and leaf endosphere, by variable selection in the rhizoplane, and by homogeneous selection in the root endosphere, which were deterministic factors leading to community succession in roots (Figure 2C). Therefore, plant niches were particularly important to microbiomes.

### 3.3 Stage also affected soybean microbiome assembly

**Figure 3:**
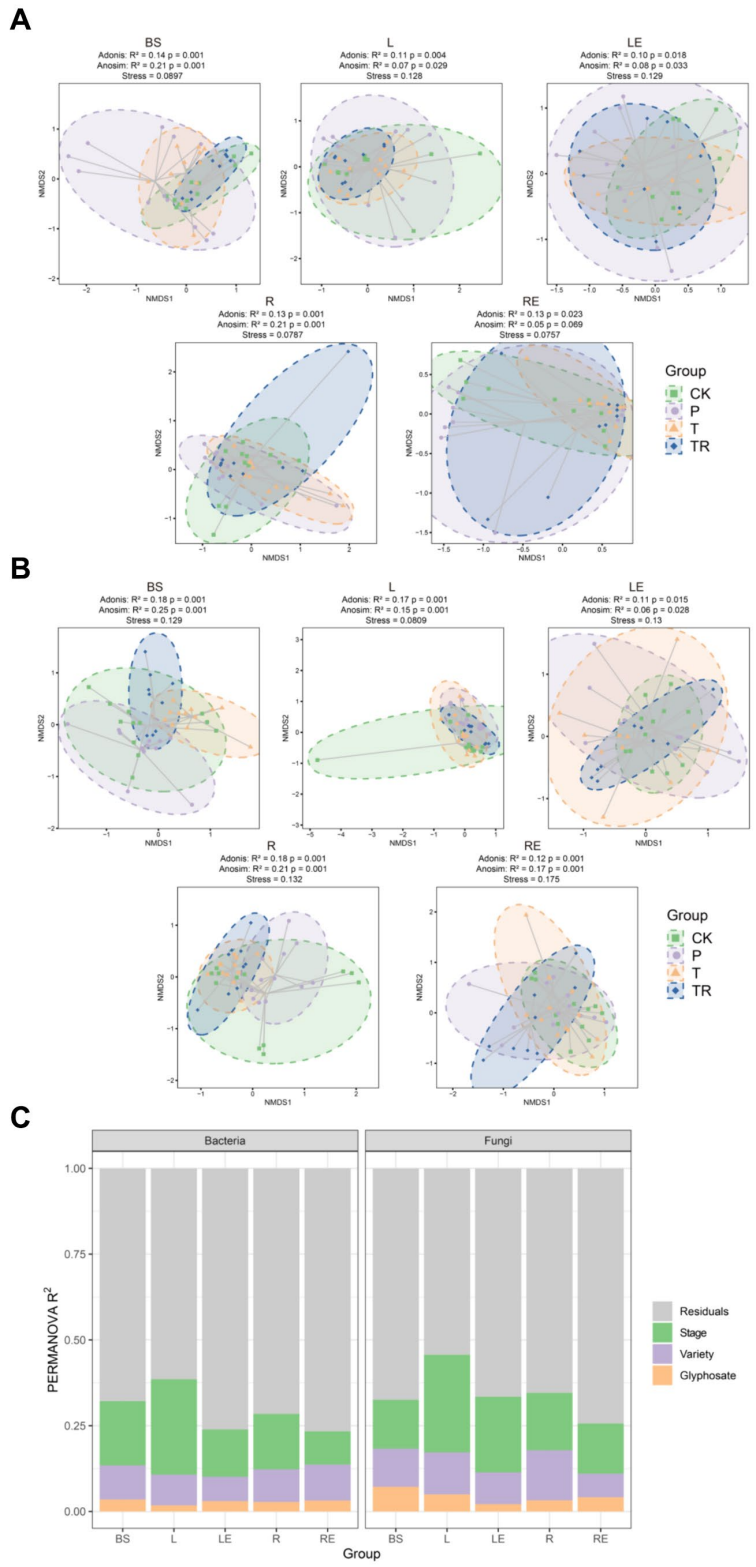
Assembly of soybean bacterial and fungal communities. Non-metric multi-dimensional scaling (NMDS) ordinations of Bray–Cutis dissimilarity matrices with permutational analysis of variance (PERMANOVA, *n* = 3, *p* < 0.05) in **(A)** bacterial and **(B)** fungal communities showing significant associations of microbial community composition with, in order of importance, the niche (bacteria: *R*^2^ = 0.43; fungi: *R*^2^ = 0.36; *p* < 0.001 for both), stage (bacteria: *R*^2^ = 0.03; fungi: *R*^2^ = 0.035; *p* < 0.001 for both), variety (bacteria: *R*^2^ = 0.005, *p* = 0.017; fungi: *R*^2^ = 0.007; *p* = 0.014), and glyphosate (bacteria: *R*^2^ = 0.003, *p* = 0.237; fungi: *R*^2^ = 0.01, *p* = 0.003). **(C)** Contributions of stage, variety, and glyphosate to the variation in bacterial (left) and fungal (right) communities in a single niche, based on PERMANOVA. Stage explained more of the variation in bacterial and fungal communities than that of variety and glyphosate in most niches. CK, conventional variety; P, conventional parental line Zhonghuang 6106; T, transgenic soybean Zhonghuang6106; TR, transgenic soybean Zhonghuang 6106 was treated with glyphosate (1 mg/L). BS, bulk soil; L, phylloplane; LE, leaf endosphere; R, rhizoplane; RE, root endosphere.

To determine the factors affecting the assembly of soybean microbiomes based on beta diversity, the contribution rates of niche, variety, transgene, stage, and glyphosate were evaluated. The NMDS ordinations and PERMANOVA analysis revealed that niche had the greatest effect on the total microbiome (bacteria: *R*^2^ = 0.429; fungi: *R*^2^ = 0.36; *p* < 0.001 for both), followed by stage (bacteria: *R*^2^ = 0.028; fungi: *R*^2^ = 0.035; *p* ≤ 0.001 for both), transgene (bacteria: *R*^2^ = 0.009; fungi: *R*^2^ = 0.013; *p* < 0.001 for both), variety (bacteria: *R*^2^ = 0.005, *p* = 0.017; fungi: *R*^2^ = 0.007, *p* = 0.014), and glyphosate (bacteria: *R*^2^ = 0.003, *p* = 0.216; fungi: *R*^2^ = 0.008, *p* = 0.004) (Figures 3A,B; Supplementary Table S3). Thus, the effects of transgene, variety, and glyphosate on the microbiomes were much less significant compared to niche and stage. For each niche, Bray–Curtis dissimilarity was applied to analyze the influence of developmental stages on bacterial and fungal communities. Bacterial communities showed more variability in the seedling stage than in other stages in bulk soil, phylloplane, rhizoplane, and root endosphere (Supplementary Table S4). Fungal communities were more variable in the mature stage than in other stages in the root endosphere (Supplementary Table S4).

To determine the effects of various factors on the microbiome during different stages, NMDS ordinations were prepared for different stages. The results showed that in soybean seedling and flowering stages (V3 and R2, respectively), variety had the greatest effect on bacterial and fungal communities. However, at the maturity stage (R8), glyphosate had the greatest effect on microbiome (Supplementary Figures S3A–H). The effect of variety on the bacterial community was lower in the root (*R^2^* = 0.029) and root endosphere (*R^2^* = 0.021) than in the bulk soil (*R^2^* = 0.041), phylloplane (*R^2^* = 0.043), and leaf endosphere (*R^2^* = 0.038) (Supplementary Table S5). Notably, the effect of variety on the fungal community was greater in the phylloplane (*R^2^* = 0.087) than in the bulk soil (*R^2^* = 0.036), leaf endosphere (*R^2^* = 0.034), rhizoplane (*R^2^* = 0.048), and root endosphere (*R^2^* = 0.041) (Supplementary Table S6), indicating that the phylloplane was more sensitive to changes in variety than the other niches.

Overall, the analysis showed that stage explained a significant proportion of variation in different niches, followed by variety, transgene, and glyphosate (Figure 3A; Supplementary Tables S5, S6). Notably, the effect of the transgene on the bacterial community was less than that of variety in the phylloplane and leaf endosphere, whereas the opposite was observed in bulk soil, rhizoplane, and root endosphere (Supplementary Tables S5, S6). The effect of the transgene on the fungal community was less than that of variety in the phylloplane and root endosphere, whereas the opposite was observed in other niches. The effect of glyphosate on bacterial communities in all parts of soybean was lower than that on fungal communities (Figures 3B,C).

### 3.4 Differences in bacterial and fungal taxa in five niches

**Figure 4:**
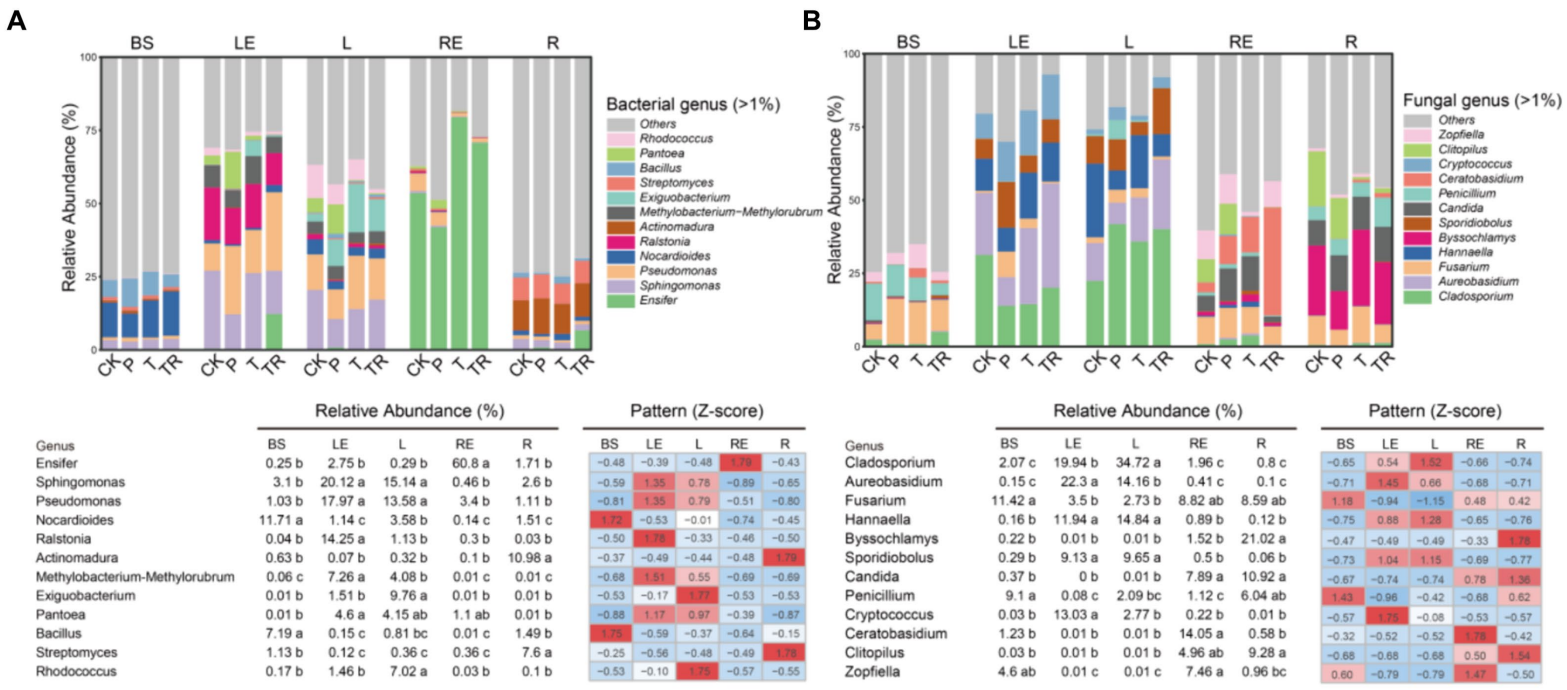
Relative abundances of core bacterial and fungal genera in five niches and four varieties. **(A)** Bacterial and **(B)** fungal genera with relative abundances >1%. To visualize the variation, relative abundances were also normalized to *Z*-score values [*Z*-score = (data point − mean)/(standard deviation)]. CK, conventional variety; P, conventional parental line of Zhonghuang 6106; T, transgenic soybean Zhonghuang 6106; TR, transgenic soybean Zhonghuang 6106 treated with glyphosate. BS, bulk soil; LE, leaf endosphere; L, phylloplane; RE, root endosphere; R, rhizoplane. Different letters indicate significant differences between niches (*n* = 3, *p* < 0.001, ANOVA).

To identify the differences in bacterial and fungal communities across the five niches, genera with relative abundance greater than 1% in each niche of each variety were screened. Among those genera, we selected the top 12 to construct stacked bar charts, relative abundance tables with indications of significant differences, and *z*-score standardized abundance heat maps (Figure 4). The abundance of *Bacillus* and *Nocardioides* was higher in bulk soil than in other niches (*p* < 0.001, ANOVA) (Figure 4A). *Pseudomonas*, *Sphingomonas*, *Methylobacterium*–*Methylorubrum*, *Ralstonia*, and *Rhodococcus* were more abundant in leaves than in BS and roots, with *Methylobacterium*–*Methylorubrum* and *Ralstonia* showing higher abundance in the leaf endosphere than in the phylloplane. The abundance of *Rhodococcus* was greater in the phylloplane than in the leaf endosphere (*p* < 0.01, ANOVA), whereas the abundance of *Ensifer* was higher in the root endosphere than in other niches (*p* < 0.01, ANOVA). *Actinomadura* and *Streptomyces* were more abundant in the rhizoplane than in other niches (*p* < 0.01, ANOVA). Furthermore, the abundance of *Exiguobacterium* was higher in the phylloplane of P, T, and TR than in that of CK (*p* < 0.01, ANOVA), and *Ensifer* was more abundant in the leaf endosphere and rhizoplane of TR than in those of other varieties (*p* < 0.001 and *p* < 0.01, respectively, ANOVA) (Figure 4A). *Pseudomonas* was more abundant in the root endosphere of CK and P than in that of T and TR.

Twelve dominant fungal genera (relative abundance ≥1.0%) were also identified. *Cladosporium*, *Aureobasidium*, *Hannaella*, and *Sporidiobolus* were more abundant in the phylloplane and leaf endosphere than in bulk soil, rhizoplane, and root endosphere (*p* < 0.01, ANOVA). The abundance of *Cryptococcus* was higher in the leaf endosphere than in other niches (*p* < 0.01, ANOVA). The abundance of *Candida* was greater in the rhizoplane and root endosphere than in bulk soil, leaf endosphere, and rhizoplane, and the abundance of *Ceratobasidium* was higher in the root endosphere than in other niches (*p* < 0.01, ANOVA). The abundance of *Byssochlamys* was higher in the rhizoplane than in other niches (*p* < 0.01, ANOVA). Moreover, the abundance of *Clitopilus* was higher in the rhizoplane and root endosphere of CK and P than in those of T and TR (*p* < 0.01, ANOVA), and *Ceratobasidium* was more abundant in the root endosphere of TR than in that of other varieties (*p* < 0.001, ANOVA) (Figure 4B).

### 3.5 Niche-affected soybean microbiome co-occurrence networks

**Figure 5:**
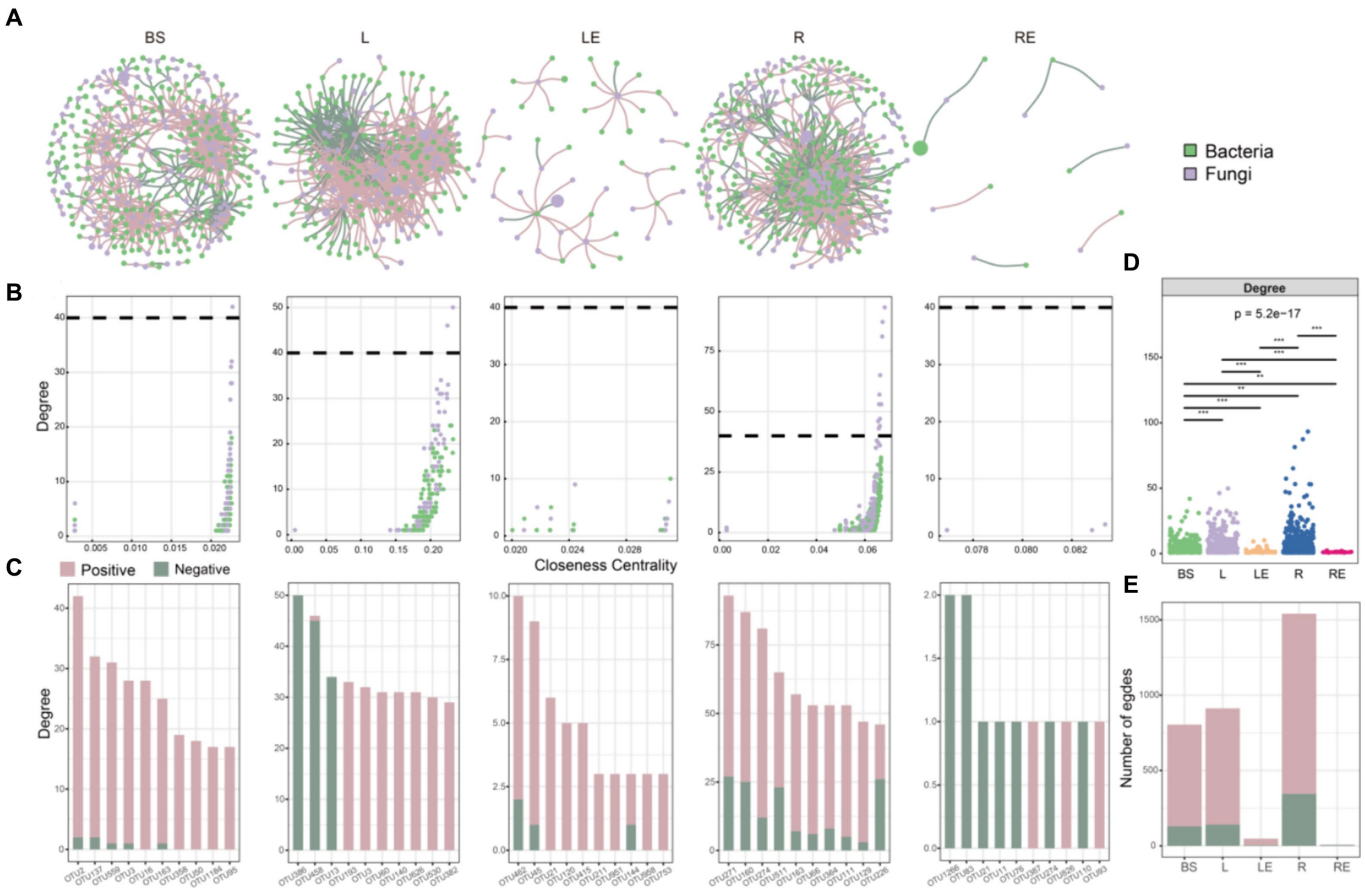
Interkingdom co-occurrence networks. **(A)** Networks containing both bacterial and fungal taxa, with a higher number of bacterial taxa (green) and a lower number of fungal taxa (purple) in bulk soil (BS), phylloplane (L), leaf endosphere (LE), rhizoplane (R), and root endosphere (RE). **(B)** Comparison of node-level topological features in figure (degree and closeness centrality) demonstrating the high degree and closeness centrality values for hub taxa. **(C)** Degree values of bacterial and fungal taxa in networks. The significance of differences was determined by non-parametric Kruskal–Wallis test. Green and amaranth colors of the edges and columns indicate positive and negative correlations, respectively. The **(D)** degree and **(E)** edges of bacterial–fungal taxa, showing network complexity in BS, L, LE, R, and RE.

We investigated how variety, transgene, and glyphosate affected soybean microbiome co-occurrence patterns in different niches. Overall, network structure was significantly affected by variety, followed by transgene and glyphosate. Based on interkingdom co-occurrence network analysis, the niche significantly affected networks (Figure 5). The highest number of nodes and negative edges was in the rhizoplane, followed by that in bulk soil, phylloplane, leaf endosphere, and root endosphere (Supplementary Table S7), indicating stronger and more stable interactions among microbiomes in the rhizoplane than in other niches. Furthermore, the edges of the top 10 hub nodes with high degree and closeness centrality values were mainly negative in the root endosphere (Figure 5; Supplementary Table S7).

The intrakingdom network analysis further indicated that co-occurrence networks were more complex and robust in CK than in P, T, and TR in all niches, as evidenced by the number of nodes and correlations (Supplementary Figure S4; Supplementary Table S7). *Proteobacteria*, *Actinobacteriota*, and *Firmicutes* were the three most abundant bacteria, and *Ascomycota*, *Basidiomycota*, and *Mucoromycota* were the three most abundant fungi (Supplementary Figure S4). In the root microbiome (including rhizoplane and root endosphere), we observed a higher proportion of positive edges and lower modularity in the bacterial networks (proportion of positive edges/modularity in roots: 99%/0.628 in CK, 99%/0.374 in P, 73%/0.459 in T, 90%/0.309 in TR) than in the fungal networks (proportion of negative edges/modularity in roots: 97%/0.689 in CK, 84%/0.766 in P, 69%/0.665 in T, 71%/0.656 in TR; Table S7), indicating that bacterial communities were less stable than fungal community in roots. We also observed a lower number of nodes and edges in the bacterial networks than in the fungal networks in bulk soil, rhizoplane, and root endosphere, whereas the opposite was observed in the leaf endosphere (Figure S4, Table S7), showing that niche affected the stability of microbiome interaction networks.

### 3.6 Source tracing of microbiomes in five niches

**Figure 6:**
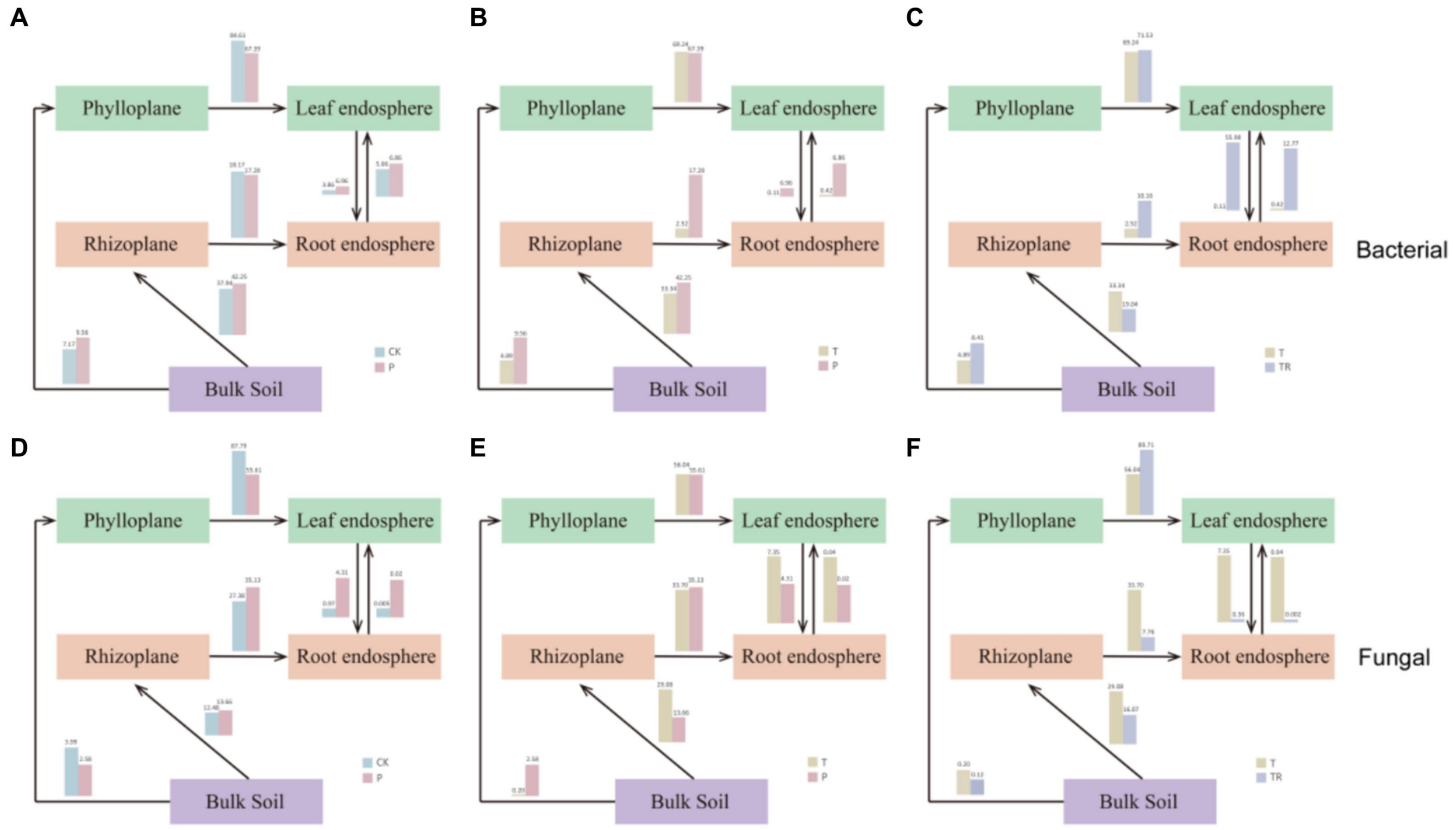
Source trace of bacterial **(A–C)** and fungal microbiomes **(D–F)**. **(A)** and **(D)**: CK VS P; **(B)** and **(E)**: T VS P; **(C)** and **(F)**: T VS TR. CK, conventional variety; P, conventional parental line of Zhonghuang 6106; T, transgenic soybean Zhonghuang 6106; TR, transgenic soybean Zhonghuang 6106 treated with glyphosate.

Analyzing the origin of bacteria and fungi in the five niches of CK, P, T, and TR allowed us to infer microbial evolution using a source trace map (Figure 6). In CK, P, T, and TR, the abundance of bacterial communities was higher than that of fungal communities from bulk soil to phylloplane (CK/P/T/TR bacteria: 7.17/9.56/4.89/8.41; fungi: 3.99/2.58/0.20/0.12). Most bacterial and fungal communities in the leaf endosphere originated significantly from the phylloplane (CK/P/T/TR bacteria: 84.61/67.39/69.24/71.53; fungi: 87.79/55.61/56.04/89.71). In CK, P, and T, approximately one-third of the bacterial communities in the rhizoplane originated from the bulk soil, with a lower proportion observed in TR (CK/P/T/TR: 37.94/42.25/33.34/19.04). The proportion of bacterial communities from the rhizoplane to the root endosphere was lower in T than in CK, P, and TR (CK/P/T/TR: 18.17/17.28/2.52/10.16). Notably, approximately one-third of the fungal microbial communities in the root endosphere originated from the rhizoplane in CK, P, and T, with a lower proportion observed in TR (CK/P/T/TR: 27.38/35.13/33.70/7.16). The bacterial communities from the leaf endosphere to the root endosphere or from the root endosphere to the leaf endosphere were highest in TR, followed by those in P and CK, and significantly lowest in T, whereas the opposite was observed in fungal communities.

## 4 Discussion

The natural microbiome affects the main functions of host plants, such as water and nutrient acquisition (Friesen et al., 2011), stress resistance (Santos-Medellín et al., 2017; Xun et al., 2021), growth promotion (Santoyo et al., 2016), and disease inhibition (Carrión et al., 2019). Therefore, the microbiome increases plant adaptability (Xun et al., 2021). In return, host plants provide habitats and sustainable energy and carbon supplies for the microbiome (Turner et al., 2013). Thus, the interactions between plants and the microbiome, as well as the factors affecting the assembly of plant-associated microbial communities, are the focus of increasing attention (Cregger et al., 2018; Moroenyane et al., 2021a; Xun et al., 2021). In soybean, the microbiome colonization pattern is regulated by the niche, growth stages of plants (Moroenyane et al., 2021a), soil type, and plant genotype (Liu et al., 2019; Liu et al., 2020b; Yang H. J. et al., 2023; Yang Q. et al., 2023).

In this study, we investigated the effects of niche, stage, variety, transgene, and glyphosate on soybean microbiomes using an amplicon approach. By profiling both bacterial and fungal communities in five below-and aboveground niches of CK, P, T, and TR plants, we revealed that fungal networks were more stable than bacterial networks in the rhizoplane (Supplementary Figure S4). Niche had the strongest influence on microbiome assembly, which was particularly evident in the rhizoplane (Figure 1). Our findings provide evidence that niche, stage, variety, transgene, and glyphosate not only changed the diversity, assembly, and networks of microbial communities but also affected their ecological functions. Notably, the effects of variety, transgene, and glyphosate were smaller than those of niche and stage. Below, we discuss how these findings advance our understanding of niche-induced changes in plant microbiome assembly, co-occurrence patterns, and functions.

### 4.1 Differences in microbial community structure across five niches and factors regulating microbiomes

Identifying factors affecting the microbiome is essential in understanding plant–microbiome interactions and in eventually utilizing microbiomes to promote agricultural sustainability (Liu J. et al., 2020; Xun et al., 2021). Our study revealed that niche exerted the greatest influence on the soybean microbiome, followed by stage, with significant differences observed among the niches, bulk soil, rhizoplane, root endosphere, phylloplane, and leaf endosphere (Figures 1, 3; Supplementary Figures S1, S3). Consistent with previous research, there were significant differences in the diversity of soybean bacterial and fungal microbiomes across plant niches (roots and leaves) (Gdanetz et al., 2021; Moroenyane et al., 2021a). Additionally, growth stage affects the composition of soybean phyllosphere (Copeland et al., 2015) and rhizosphere microbial communities (Xu et al., 2009; Sugiyama et al., 2014). These findings indicated that the composition of bacterial and fungal microbial communities varies widely among different niches, indicating that tissue specificity is a powerful driving force for microbial community successional patterns, as observed in a previous study on poplar (Cregger et al., 2018).

Sampling soybean niches at different growth stages allowed seasonal trends affecting microbial community abundance and diversity to be identified. The Shannon diversity index, Bray–Curtis dissimilarity, and NMDS analysis showed significant differences in diversity within the same niche across different growth stages, with different growth stages affecting bacterial and fungal communities differently within the same niche (Supplementary Figures S1, S3; Supplementary Table S4). The abundance and composition of plant exudates released at each growth stage can affect microbial successional patterns in the rhizosphere (Philippot et al., 2013; White et al., 2015). We postulate that the metabolites exchanged between soybean and microbiomes at different growth stages were different. Moreover, bacterial diversity in the leaf endosphere was lower than that in the phylloplane at different stages, and bacterial diversity in the root endosphere was lower than that in the rhizoplane (Figures 1, 3; Supplementary Figures S1, S3), indicating that the plant internal environment was more stable than the external environment (Figure 2; Supplementary Figure S2). Ecological drift induces random fluctuations in abundance, reducing diversity and leading to differentiation of community structure (Gilbert and Levine, 2017). Hence, the changes in this study might result from ecological drift, and interactions between niches and microbiome at different growth stages May lead to independent microbial niches.

Transgene and glyphosate had lower effects on the microbiome compared with niches and genotypes (Supplementary Table S3). CK, P, and T represented different soybean genotypes and consistent with previous studies, indicated that the influence of plant genotype on assembly of the rhizosphere microbiome was usually weak, which can vary depending on environmental factors and plant characteristics (Pérez-Jaramillo et al., 2016). However, although genotype does not seem to significantly affect microbial community composition, genes involved in immune, nutritional, and stress responses May alter the structure of specific microbiomes and thereby profoundly affect host performance (Zhou et al., 2022; Timm et al., 2018; Castrillo et al., 2017).

### 4.2 Differences in the variation of core microbial taxa in relation to different niche-specific functions

Core bacterial and fungal communities vary across different ecological niches (Yang H. J. et al., 2023; Yang Q. et al., 2023). The interaction of plant biochemical products May be jointly regulated by the selection of microbial members within organ niches (Yang H. J. et al., 2023; Yang Q. et al., 2023). In our study, the core microbes in different niches were indeed different (Figure 4). The influence of different ecological niches on the core microbiome is mainly due to environmental factors or plant–microbial interactions. For example, microorganisms in leaves are affected by air, light, and rainfall (Zhan et al., 2022), whereas those in roots are mainly affected by soil physical and chemical properties, such as pH, nutrient availability, and moisture and temperature, which shape the structure of soil microbial communities (Bahram et al., 2018; van den Hoogen et al., 2019). Root exudates such as triterpenoids (Huang et al., 2019), benzoxazines (Hu et al., 2018), and other organic compounds (Eilers et al., 2010) are also important driving forces for rhizosphere microbiome assembly.

The microbial genera *Bacillus, Pseudomonas, Streptomyces, Cladosporium* (Zhou et al., 2022), *Nocardioides* (Yu et al., 2023), *Sphingomonas* (Shi L. et al., 2024), *Methylobacterium*–*Methylorubrum* (Xie et al., 2023), *Rhodococcus* (Akram et al., 2024; Wu et al., 2024), *Ensifer* (Hao et al., 2024), *Actinomadura* (Zhang et al., 2024), *Hannaella* (Yang H. J. et al., 2023; Yang Q. et al., 2023), *Sporidiobolus* (Wang et al., 2023), and *Ceratobasidium* (Shi Z. et al., 2024) were significantly enriched, and those genera have the potential to promote plant growth or biological control functions. For example, *Cladosporium* is a pathogen-suppressing microbe (Mendes et al., 2011; Lakshmeesha et al., 2020). The microbiomes dominated by such genera might have different roles in the maintenance of ecosystems in different niches and identifying the different roles would deepen our understanding of microbial protection of plants from pathogen invasion.

Typically, relatively high microbial diversity increases network complexity (Qiu et al., 2022). After spraying glyphosate, the core species of bacteria in the root endosphere decreased significantly compared with other niches, and network stability also decreased; however, fungi were not similarly affected (Figure 4; Supplementary Table S7). The network stability of different ecological niches also varied significantly. The types and abundance of the dominant microbes in the root endosphere were relatively low, and network stability in that niche was also low (Figure 5; Supplementary Figure S4). By contrast, the types and quantities of dominant microbes in the rhizoplane were relatively high, resulting in increased network stability (Figure 4; Supplementary Figure S4). These results indicate that stability of microbial community networks May be closely related to the number of core taxonomic groups in a community.

The microbiome in the leaf endosphere was greatly affected by that in the phylloplane, whereas the root endosphere was relatively less affected by the rhizoplane (Figure 6). We hypothesized that compared with a leaf, the soil environment was more complex and there were more factors affecting bacterial communities in the root endosphere. In addition, NCM analysis also showed that roots were subject to more selection than leaves (Supplementary Figure S2). There is extensive taxonomic overlap between leaf and root microbial communities, with a significant overlap in the genome-encoded functional abilities of leaf-derived and root-derived bacteria and little difference in individual functional categories (Bai et al., 2015). Source tracing of bacterial and fungal microbiomes showed a significant proportion of overlap between leaf and root microbial communities (Figure 6), indicating the potential for mutual migration between root and leaf microbial communities.

## 5 Conclusion

By focusing on the changes in soybean-associated bacterial and fungal microbiomes of different soybean varieties across different niches and stages, we found significant differences in the soybean microbiome among different niches, with different stages also significantly affecting microbial diversity. Variety, transgene, and glyphosate had little effect on microbial communities, with glyphosate having the lowest effect. Dominant genera and core microbial communities differed across the five niches, likely due to plant–microbial interactions. The core microbial communities were dominated by beneficial microbes that might promote plant growth or increase disease resistance. Thus, our results May help develop plant protection technologies that utilize microbiomes for biological control of diseases.

## Data Availability

The datasets presented in this study can be found in online repositories. The names of the repository/repositories and accession number(s) can be found below: https://www.ncbi.nlm.nih.gov/, PRJNA1092852.

## Supplementary material

SUPPLEMENTARY FIGURE 1 Bacterial and fungal communities in different niches at different stages in soybean. Alpha diversity of **(A–D)** bacterial and **(E–H)** fungal communities in different niches in different soybean lines (one-way ANOVA, *n* = 3). *, ** and *** significant at 0.05, 0.01 and 0.001 probability levels, respectively. CK: conventional variety; P: conventional parental line Zhonghuang6106; T: transgenic soybean Zhonghuang6106; TR: Transgenic soybean Zhonghuang6106 was treated withglyphosate (1 mg/L). BS: bulk soil; L: phylloplane; LE: leaf endosphere; R: rhizoplane; RE: root endosphere. V3: seedling stage; R2: flowering stage; R5: podding stage; R8: maturity stage.

Supplementary FIGURE 2 Fit of the neutral community model (NCM) of bacteria and fungi community assembly. Predicted occurrence frequencies for bacteria in **(A)** bulk soil (BS), **(B)** phylloplane (L), **(C)** leaf endosphere (LE), **(D)** rhizoplane (R) and **(E)** root endosphere (RE). The predicted occurrence frequencies for fungi in **(F)** BS, **(G)** L, **(H)** LE, **(I)** R and **(J)** RE respectively. Solid blue lines indicate the best fit to the NCM, and dashed blue lines represent 95% confidence intervals around the model prediction. ASVs that occur more or less frequently than predicted by the NCM are shown in different colors. Nm indicates the metacommunity size times immigration; Rsqr indicates the fit to NCM.

Supplementary FIGURE 3 Assembly of soybean bacterial and fungal communities in different stages. Non-metric multi-dimensional scaling (NMDS) ordinations of Bray–Cutis dissimilarity matrices with permutational analysis of variance (PERMANOVA, *n* = 3, *P* < 0.05), showing significant association of the bacterial community composition in **(A)** V3 stage, **(B)** R2 stage, **(C)** R5 stage, **(D)** R8 stage. NMDS ordinations of Bray–Cutis dissimilarity matrices with PERMANOVA showing significant association of the fungal community composition in **(E)** V3 stage, **(F)** R2 stage, **(G)** R5 stage, **(H)** R8 stage. V3: seedling stage; R2: flowering stage; R5: podding stage; R8: maturity stage. CK: conventional variety; P: conventional parental line Zhonghuang6106; T: transgenic soybean Zhonghuang6106; TR: Transgenic soybean Zhonghuang6106 was treated withglyphosate (1 mg/L). BS: bulk soil; L: phylloplane; LE: leaf endosphere; R: rhizoplane; RE: root endosphere.

Supplementary FIGURE 4 Intra-kingdom co-occurrence networks. **(A)** Intra-kingdom co-occurrence networks of bacteria in bulk soil (BS). **(B)** Intra-kingdom co-occurrence networks of bacteria in phylloplane (L). **(C)** Intra-kingdom co-occurrence networks of bacteria in leaf endosphere (LE). **(D)** Intra-kingdom co-occurrence networks of bacteria in rhizoplane (R). **(E)** Intra-kingdom co-occurrence networks of bacteria in root endosphere (RE). **(F)** Intra-kingdom co-occurrence networks of fungi in BS. **(G)** Intra-kingdom co-occurrence networks of fungi in L. **(H)** Intra-kingdom co-occurrence networks of fungi in LE. **(I)** Intra-kingdom co-occurrence networks of fungi in R. **(J)** Intra-kingdom co-occurrence networks of fungi in RE. The nodes are colored according to bacterial phylum and fungal class. Node size indicates the degree of connection. Green and amaranth colors of the edges and columns indicate positive and negative correlations, respectively. Taxonomic information for hub taxa is presented in Supplementary Table S7. Degree and interaction type of the top 10 hub nodes in four networks. The degree and edges of bacterial and fungal taxa showing network complexity in different soybeans. The significance of difference was determined by nonparametric Kruskal–Wallis test.
