# Supplementary material for: The response of microbiome assembly within different niches across four stages to the cultivation of glyphosate-tolerant and conventional soybean varieties
Source: Front Microbiol. 2024 Sep 25;15:1439735. doi: 10.3389/fmicb.2024.1439735 (PMC11461410; doi:10.3389/fmicb.2024.1439735)
Supplement: Supplementary file 6 [file Table_2.DOCX]

**Table S1 Overview of 16S amplicon data**

|  | **Sequencing Samples** | **Number of Raw Reads** | | | **Number of Clean Reads** | | **Number of ASVs** | | **Primer** |
| --- | --- | --- | --- | --- | --- | --- | --- | --- | --- |
|  |  | **Total** | **Minimum** | **Maximum** | **Non_chimeric and merged** | **Microbial reads** | **Total** | **Assigned ASVs** |  |
| **Bacteria** | n=240 | 14,348,482 | 32,911 | 79,598 | 11,682,876 | 11,682,876 | 81,790 | 81,482 | 799F(5'-AACMGGATTA GTAGATACCCKG-3') and 1193R(5'-ACGTCATCCCCACCTTC C-3') |
| **Fungi** | n=240 | 15,700,058 | 35,970 | 74,949 | 15,613,314 | 15,613,314 | 13,321 | 10,129 | ITS1F(5'-CTTGGTCATTTAGAGGAAGTAA-3') and ITS2R(5'-GCTGCGTTCTTCATCGATGC-3') |

**Table S2 Linear-mixed models (LMMs) for alpha diversity indices. Effects of niche, variety, stage and glyphosate on bacterial and fungal community alpha diversity indices were tested with linear-mixed models (LMMs). Significance was assessed using type II ANOVA with Kenward–Rodger approximation of the degrees of freedom in a linear-mixed model.**

| Microbial  Communities | Variables | Shannon Diversity | | Chao1 richness | |
| --- | --- | --- | --- | --- | --- |
|  |  | F value | P (> F) | F value | P (> F) |
| Bacterial Community | Compartment | 166.54 | 7.58E-65 | 509.08 | 5.61E-109 |
|  | Variety | 3.38 | 0.067 | 4.31 | 0.040 |
|  | Transgene | 0.23 | 0.636 | 3.74 | 0.056 |
|  | Stage | 1.39 | 0.246 | 0.81 | 0.489 |
|  | Glyphosate | 0.00 | 0.963 | 0.31 | 0.580 |
| Fungal  Community | Compartment | 43.70 | 7.04E-27 | 318.20 | 6.60E-89 |
|  | Variety | 0.31 | 0.580 | 1.75 | 0.188 |
|  | Transgene | 0.25 | 0.637 | 13.25 | 0.001 |
|  | Stage | 3.32 | 0.021 | 2.83 | 0.039 |
|  | Glyphosate | 0.12 | 0.729 | 0.56 | 0.455 |

**Table S3 PERMANOVA by adonis of all bacterial 16S and fungal ITS samples. PERMANOVA analysis using the Bray Curtis distances for niche, variety, stage and glyphosate in beta diversity.**

| **Microbial  Communities** | **Variables** | **Df** | **SumsOfSqs** | **MeanSqs** | **F.Model** | ***R*^2^** | **Pr(>F)** |
| --- | --- | --- | --- | --- | --- | --- | --- |
| **Bacterial Community** | Compartment | 4 | 38.26 | 9.56 | 43.64 | 0.429 | 0.001 |
|  | Stage | 3 | 2.48 | 0.83 | 3.77 | 0.028 | 0.001 |
|  | Variety | 1 | 0.47 | 0.47 | 2.14 | 0.005 | 0.017 |
|  | Transgene | 1 | 0.80 | 0.80 | 3.66 | 0.009 | 0.001 |
|  | Glyphosate | 1 | 0.27 | 0.27 | 1.23 | 0.003 | 0.216 |
| **Fungal  Community** | Compartment | 4 | 30.95 | 7.74 | 33.24 | 0.360 | 0.001 |
|  | Stage | 3 | 2.99 | 1.00 | 4.28 | 0.035 | 0.001 |
|  | Variety | 1 | 0.57 | 0.57 | 2.43 | 0.007 | 0.014 |
|  | Transgene | 1 | 1.09 | 1.09 | 4.67 | 0.013 | 0.001 |
|  | Glyphosate | 1 | 0.67 | 0.67 | 2.89 | 0.008 | 0.004 |

Df: degrees of freedom, SumsOfSqs: sum of squares, MeanSqs: mean sum of squares, Pr: p-values are based on 999 permutations with subsequent Bonferroni correction.

**Table S4 Distance to centroid was calculated by analysis of beta dispersion using Bray-Curtis dissimilarity.**

| **Community Dissimilarity** | | **Bacterial** | | **Fungal** | |
| --- | --- | --- | --- | --- | --- |
| **Niche** | **Stage** | **Average Distance to Centriod** | **Pr(>F)** | **Average Distance to Centriod** | **Pr(>F)** |
| **BS** | S | 0.3835 | 0.012* | 0.4867 | 0.412 |
|  | F | 0.3503 |  | 0.5104 |  |
|  | B | 0.3289 |  | 0.4501 |  |
|  | M | 0.3372 |  | 0.4835 |  |
| **L** | S | 0.5122 | 0.031* | 0.3853 | 0.051 |
|  | F | 0.4005 |  | 0.2966 |  |
|  | B | 0.4377 |  | 0.2586 |  |
|  | M | 0.4325 |  | 0.2697 |  |
| **LE** | S | 0.5051 | 0.752 | 0.3477 | 0.479 |
|  | F | 0.4866 |  | 0.3959 |  |
|  | B | 0.5251 |  | 0.4327 |  |
|  | M | 0.4884 |  | 0.3912 |  |
| **R** | S | 0.4057 | 0.002** | 0.4582 | 0.175 |
|  | F | 0.3346 |  | 0.4334 |  |
|  | B | 0.3712 |  | 0.3649 |  |
|  | M | 0.3092 |  | 0.4256 |  |
| **RE** | S | 0.5027 | 0.231 | 0.4818 | 0.001*** |
|  | F | 0.4012 |  | 0.5561 |  |
|  | B | 0.295 |  | 0.5661 |  |
|  | M | 0.4884 |  | 0.6098 |  |

**Table S5 PERMANOVA by adonis of bacterial 16S conducted separately for each niche. PERMANOVA analysis using the BrayCurtis distances for variety, stage and glyphosate performed separately at individual niche.**

| **Niche** | **Factor** | **Df** | **SumsOfSqs** | **MeanSqs** | **F.Model** | ***R*^2^** | **Pr(>F)** |
| --- | --- | --- | --- | --- | --- | --- | --- |
| **BS** | Stage | 3 | 1.31 | 0.44 | 3.60 | 0.193 | 0.001 |
|  | Variety | 1 | 0.28 | 0.28 | 2.29 | 0.041 | 0.001 |
|  | Transgene | 1 | 0.36 | 0.36 | 2.97 | 0.053 | 0.001 |
|  | Glyphosate | 1 | 0.24 | 0.24 | 1.96 | 0.035 | 0.002 |
| **L** | Stage | 3 | 3.57 | 1.19 | 5.82 | 0.282 | 0.001 |
|  | Variety | 1 | 0.54 | 0.54 | 2.67 | 0.043 | 0.002 |
|  | Transgene | 1 | 0.53 | 0.53 | 2.58 | 0.042 | 0.005 |
|  | Glyphosate | 1 | 0.23 | 0.23 | 1.10 | 0.018 | 0.306 |
| **LE** | Stage | 3 | 1.89 | 0.63 | 2.33 | 0.140 | 0.001 |
|  | Variety | 1 | 0.52 | 0.52 | 1.92 | 0.038 | 0.027 |
|  | Transgene | 1 | 0.41 | 0.41 | 1.53 | 0.031 | 0.109 |
|  | Glyphosate | 1 | 0.41 | 0.41 | 1.49 | 0.030 | 0.075 |
| **R** | Stage | 3 | 1.06 | 0.35 | 2.77 | 0.156 | 0.001 |
|  | Variety | 1 | 0.19 | 0.19 | 1.52 | 0.029 | 0.043 |
|  | Transgene | 1 | 0.49 | 0.49 | 3.82 | 0.072 | 0.001 |
|  | Glyphosate | 1 | 0.19 | 0.19 | 1.46 | 0.028 | 0.070 |
| **RE** | Stage | 3 | 1.22 | 0.41 | 1.81 | 0.110 | 0.049 |
|  | Variety | 1 | 0.24 | 0.24 | 1.06 | 0.021 | 0.328 |
|  | Transgene | 1 | 0.79 | 0.79 | 3.51 | 0.071 | 0.011 |
|  | Glyphosate | 1 | 0.35 | 0.35 | 1.57 | 0.032 | 0.145 |

Note: Df: degrees of freedom, SumsOfSqs: sum of squares, MeanSqs: mean sum of squares, Pr: p-values are based on 999 permutations with subsequent Bonferroni correction.

**Table S6 PERMANOVA by adonis of fungal ITS conducted separately for each niche. PERMANOVA analysis using the Bray Curtis distances for variety, stage and glyphosate performed separately at individual niche.**

| **Compartment** | **Factor** | **Df** | **SumsOfSqs** | **MeanSqs** | **F.Model** | **R2** | **Pr(>F)** |
| --- | --- | --- | --- | --- | --- | --- | --- |
| **BS** | Stage | 3 | 1.80 | 0.60 | 2.70 | 0.144 | 0.001 |
|  | Variety | 1 | 0.45 | 0.45 | 2.01 | 0.036 | 0.015 |
|  | Transgene | 1 | 0.93 | 0.93 | 4.16 | 0.074 | 0.001 |
|  | Glyphosate | 1 | 0.90 | 0.90 | 4.05 | 0.072 | 0.001 |
| **L** | Stage | 3 | 1.77 | 0.59 | 6.61 | 0.284 | 0.001 |
|  | Variety | 1 | 0.54 | 0.54 | 6.06 | 0.087 | 0.001 |
|  | Transgene | 1 | 0.23 | 0.23 | 2.56 | 0.037 | 0.029 |
|  | Glyphosate | 1 | 0.31 | 0.31 | 3.47 | 0.050 | 0.007 |
| **LE** | Stage | 3 | 2.19 | 0.73 | 4.27 | 0.224 | 0.001 |
|  | Variety | 1 | 0.33 | 0.33 | 1.96 | 0.034 | 0.050 |
|  | Transgene | 1 | 0.53 | 0.53 | 3.11 | 0.054 | 0.001 |
|  | Glyphosate | 1 | 0.21 | 0.21 | 1.22 | 0.021 | 0.260 |
| **R** | Stage | 3 | 1.69 | 0.56 | 3.31 | 0.171 | 0.001 |
|  | Variety | 1 | 0.48 | 0.48 | 2.79 | 0.048 | 0.002 |
|  | Transgene | 1 | 0.93 | 0.93 | 5.48 | 0.094 | 0.001 |
|  | Glyphosate | 1 | 0.32 | 0.32 | 1.88 | 0.032 | 0.030 |
| **RE** | Stage | 3 | 2.46 | 0.82 | 2.51 | 0.148 | 0.001 |
|  | Variety | 1 | 0.68 | 0.68 | 2.10 | 0.041 | 0.002 |
|  | Transgene | 1 | 0.44 | 0.44 | 1.34 | 0.026 | 0.075 |
|  | Glyphosate | 1 | 0.69 | 0.69 | 2.12 | 0.041 | 0.002 |

**Table S7 Topology properties of the inter- and intrakingdom networks.**

| **Network** | **No.nodes** | **No.positive edges /proportion(%)** | **No.negative edges /proportion(%)** | **clustering coefficient** | **Avg. Degree** | **Modularity** | **Network density** |
| --- | --- | --- | --- | --- | --- | --- | --- |
| **Interkindom_BS** | 368 | 675/84% | 130/16% | 0 | 10.208 | 0.679 | 0.012 |
| **Interkindom_L** | 225 | 772/85% | 141/15% | 0 | 22.493 | 0.466 | 0.036 |
| **Interkindom_LE** | 51 | 44/92% | 4/8% | 0 | 5.107 | 0.752 | 0.038 |
| **Interkindom_R** | 372 | 1196/78% | 345/22% | 0 | 25.46 | 0.352 | 0.022 |
| **Interkindom_RE** | 14 | 2/25% | 6/75% | 0 | 1.286 | 0.813 | 0.088 |
| **Intra_Bacterial_BS_CK** | 101 | 252/85% | 44/15% | 0.477 | 7.102 | 0.634 | 0.059 |
| **Intra_Bacterial_BS_P** | 70 | 173/76% | 54/24% | 0.587 | 7.679 | 0.6 | 0.094 |
| **Intra_Bacterial_BS_T** | 67 | 106/92% | 9/8% | 0.529 | 4.148 | 0.714 | 0.052 |
| **Intra_Bacterial_BS_TR** | 67 | 255/83% | 52/17% | 0.624 | 10.223 | 0.472 | 0.139 |
| **Intra_Bacterial_L_CK** | 91 | 426/98% | 7/2% | 0.567 | 11.046 | 0.5 | 0.106 |
| **Intra_Bacterial_L_P** | 90 | 368/92% | 30/8% | 0.541 | 10.985 | 0.429 | 0.099 |
| **Intra_Bacterial_L_T** | 63 | 113/95% | 6/5% | 0.794 | 4.295 | 0.591 | 0.061 |
| **Intra_Bacterial_L_TR** | 66 | 78/64% | 43/36% | 0.419 | 4.485 | 0.656 | 0.056 |
| **Intra_Bacterial_LE_CK** | 89 | 165/79% | 45/21% | 0.481 | 6.038 | 0.63 | 0.054 |
| **Intra_Bacterial_LE_P** | 85 | 215/96% | 9/4% | 0.612 | 6.184 | 0.702 | 0.063 |
| **Intra_Bacterial_LE_T** | 73 | 112/89% | 14/11% | 0.541 | 4.187 | 0.736 | 0.048 |
| **Intra_Bacterial_LE_TR** | 49 | 96/83% | 19/17% | 0.546 | 5.597 | 0.616 | 0.098 |
| **Intra_Bacterial_R_CK** | 79 | 265/99% | 1/1% | 0.611 | 7.861 | 0.628 | 0.086 |
| **Intra_Bacterial_R_P** | 61 | 274/99% | 1/1% | 0.593 | 10.88 | 0.374 | 0.15 |
| **Intra_Bacterial_R_T** | 68 | 193/73% | 70/27% | 0.652 | 9.284 | 0.459 | 0.115 |
| **Intra_Bacterial_R_TR** | 50 | 248/90% | 28/10% | 0.634 | 12.598 | 0.309 | 0.225 |
| **Intra_Bacterial_RE_CK** | 84 | 766/99% | 2/1% | 0.712 | 21.263 | 0.302 | 0.22 |
| **Intra_Bacterial_RE_P** | 55 | 572/98% | 12/2% | 0.763 | 22.86 | 0.166 | 0.393 |
| **Intra_Bacterial_RE_T** | 41 | 135/99% | 2/1% | 0.636 | 7.679 | 0.444 | 0.167 |
| **Intra_Bacterial_RE_TR** | 43 | 97/94% | 6/6% | 0.651 | 5.785 | 0.428 | 0.114 |
| **Intra_Fungal_BS_CK** | 135 | 353/90% | 38/10% | 0.531 | 7.375 | 0.579 | 0.043 |
| **Intra_Fungal_BS_P** | 111 | 201/94% | 12/6% | 0.544 | 4.567 | 0.793 | 0.035 |
| **Intra_Fungal_BS_T** | 94 | 174/99% | 2/1% | 0.466 | 4.816 | 0.736 | 0.04 |
| **Intra_Fungal_BS_TR** | 132 | 467/68% | 216/32% | 0.534 | 12.568 | 0.585 | 0.079 |
| **Intra_Fungal_L_CK** | 112 | 505/99% | 3/1% | 0.933 | 9.641 | 0.621 | 0.082 |
| **Intra_Fungal_L_P** | 70 | 127/74% | 44/26% | 0.518 | 6.011 | 0.535 | 0.071 |
| **Intra_Fungal_L_T** | 65 | 112/90% | 12/10% | 0.574 | 4.478 | 0.582 | 0.06 |
| **Intra_Fungal_L_TR** | 39 | 79/52% | 72/48% | 0.621 | 9.834 | 0.273 | 0.204 |
| **Intra_Fungal_LE_CK** | 56 | 75/87% | 11/13% | 0.793 | 3.363 | 0.732 | 0.056 |
| **Intra_Fungal_LE_P** | 56 | 139/71% | 56/29% | 0.561 | 8.309 | 0.398 | 0.127 |
| **Intra_Fungal_LE_T** | 49 | 115/99% | 1/1% | 0.82 | 5.49 | 0.627 | 0.099 |
| **Intra_Fungal_LE_TR** | 41 | 97/57% | 72/43% | 0.759 | 9.765 | 0.239 | 0.206 |
| **Intra_Fungal_R_CK** | 94 | 368/97% | 13/3% | 0.673 | 9.048 | 0.689 | 0.087 |
| **Intra_Fungal_R_P** | 84 | 145/84% | 28/16% | 0.666 | 4.714 | 0.766 | 0.05 |
| **Intra_Fungal_R_T** | 119 | 267/69% | 121/31% | 0.567 | 8.101 | 0.665 | 0.055 |
| **Intra_Fungal_R_TR** | 99 | 288/71% | 120/29% | 0.618 | 9.854 | 0.656 | 0.084 |
| **Intra_Fungal_RE_CK** | 96 | 118/97% | 4/3% | 0.731 | 2.841 | 0.878 | 0.027 |
| **Intra_Fungal_RE_P** | 91 | 93/96% | 4/4% | 0.681 | 2.394 | 0.906 | 0.024 |
| **Intra_Fungal_RE_T** | 119 | 126/94% | 8/6% | 0.592 | 2.625 | 0.887 | 0.019 |
| **Intra_Fungal_RE_TR** | 78 | 227/84% | 44/16% | 0.782 | 7.828 | 0.603 | 0.09 |
